# Toxicological assessment of the hydroethanolic leaf extract of *clerodendrum capitatum* in Wistar rats

**DOI:** 10.11604/pamj.2016.24.66.8771

**Published:** 2016-05-16

**Authors:** Kokou Idoh, Amegnona Agbonon, Yao Potchoo, Messanvi Gbeassor

**Affiliations:** 1 Laboratory of Physiology, Pharmacology and Toxicology, Faculty of Sciences, University of Lomé, Lomé, Togo; 2 Laboratory of Pharmacology, Department of Pharmacy, Faculty of Health Sciences, University of Lomé, Lomé, Togo

**Keywords:** Clerodendrum capitatum, oral toxicity, safety, Wistar rats

## Abstract

**Introduction:**

*Clerodendrum capitatum* (Willd) Schumach. & Thonn (Lamiaceae) is used in African traditional medicine for the treatment of malaria, hypertension, obesity, jaundice and diabetes however there is lack of experimental data on its possible toxicity. This study investigated the acute and 28 days sub-chronic toxicity of *C. capitatum* in Wistar rats.

**Methods:**

In acute toxicity tests, a single administration of the hydroethanolic *C. capitatum* leaf extract (5 g/kg) was given orally to 5 female rats. The general behavior, adverse effects and mortality were recorded for up to 14 days post treatment. On the 15^th^ day, the rats were weighed and euthanized for necropsy. In sub-chronic toxicity tests, the extract (4, 8 and 16 g/kg/day) was given orally to both male and female rats for 28 days. The animal body weight was recorded throughout the experiment, while hematological and biochemical parameters of blood and relative organs weights were evaluated on the 29^th^ day.

**Results:**

*Clerodendrum Capitatum* did not cause any death or any hazardous symptoms of acute toxicity, showing an LD_50_ higher than 5 g/kg. Sub-chronic administration of C. capitatum resulted in no noticeable changes in weight gain and water or food consumption. White blood cells and hemoglobin increased while urea concentration, liver enzymes, total cholesterol and glucose concentrations significantly decreased in treated animals. No changes in macroscopical aspect of organs were observed in the animals.

**Conclusion:**

These results showed that acute or sub-chronic oral administration of the hydroethanolic leaf extract of Clerodendrum capitatum may be considered as relatively free of toxicity.

## Introduction

The use of medicinal plants to treat various ailments is as old as the world. It is well known throughout history and has always been part of human culture. The World Health Organization (WHO) estimates that up to 80% of the world's population relies on traditional medicinal system for some aspects of primary health care [1]. In African ethnomedicines, it is well known that traditional healers make use of a large variety of herbs in the treatment of parasitic diseases including malaria and a wide proportion of herbal remedies dispensed by traditional healers are widely believed by their clients to be effective [2, 3]. Such plants can play an important role in drug discovery and their studies are logical research strategies in the search for new drugs [4]. In some countries, government encourages the use of indigenous forms of medicine rather than expensive imported drugs. In the USA, nearly one in three people uses some kind of alternative medical treatment [5]. Previous studies have reported the effectiveness of medicinal plants in treating of various disease conditions such as liver problems [6], circulatory and respiratory problems [7], febrile illnesses [8] and diarrhea [9]. However, despite the profound therapeutic advantages possessed by some of the plants, some constituents of medicinal plants have been shown to be potentially toxic, carcinogenic and teratogenic [10, 11]. Moreover, it is known that the consumption of medicinal plants without evaluating their efficacy and safety can result in unexpected or toxic effects that may affect the physiology of different organs in the human body. Liver and kidney are the first targets in toxicological evaluation because they are involved in the metabolism and excretion of chemical compounds. Renal damage has also been associated with the use of medicinal plants in the treatment of various diseases [12].

*Clerodendrum capitatum* (Willd) Schumach. & Thonn (Lamiaceae) is an indigenous tropical Africa perennial undershrub, fast growing, erect, well branched, and grows up to 0.5-2 m high [13]. In Togo the leaves of the plant are traditionally used in the treatment of malaria [14]. Based on ethno botanical report in the maritime region of Togo, *C. capitatum* is frequently used to treat high blood pressure related to hypertension and also to alleviate obesity, jaundice and constipation. In Nigeria, the plant is used to treat diabetes mellitus, obesity and high blood pressure [15]. Pharmacological studies of the *Clerodendrum* genus demonstrated biological activities such as antitumorgenic [16, 17], hypoglycemic, and hypolipidemic [18]. Despite the widespread use of *C. capitatum*, there is lack of experimental data on its possible toxicity. Thus, the present investigation was undertaken to provide data on the safety of *C. capitatum* by focusing on the acute toxicity and 28 days sub-chronic toxicity of the hydroethanolic leaf extract of *C. capitatum* given orally to male and female Wistar rats.

## Methods

### Plant material and extraction

Fresh leaves of *C. capitatum* were collected in August 2013 from Adidigomé, a locality at North West of Lomé (Togo). Botanical authentication was confirmed at the Department of Botany, University of Lomé, where a voucher specimen of *C. capitatum* was deposited at the herbarium (N° TG12806). The leaves were washed, air-dried under air conditioner for three days and were coarsely powdered. The powder (300 g) was macerated at room temperature with 3 L of ethanol-water (8:2, v/v) for 72 h. The solvent was removed using a rotary vacuum evaporator (Buchi, Japan) at 40°C and the crude extract, representing a yield of approximately 12.7% (w/w), was stored at -4°C until administrated doses preparation, when it was dissolved in sterilized distilled water.

### Animals and treatment

Wistar rats of either sex weighting 145-150 g (males) and 120-125 g (females) used in the present study were provided by animals facilities of the Laboratory of Physiology, Pharmacology and toxicology of the Faculty of Sciences, University of Lomé. Animals were housed divided by sex in cages (Five rats per cage) at ambient temperature and humidity with a 12 hours day-light cycle, with free access to food and water ad *libitum*. Experimental protocols were based on World Health Organization Guidelines for care and use of laboratory animals, and the use of the animals was approved by the Ethics Committee of the University of Lomé, a branch of the National Ethics Committee for control and supervision of experiments on animals (N° SBM/UL/14/NS0004).

### Acute toxicity

Healthy female Wistar rats were used in this study according to the Organization for Economic Cooperation and Development (OECD) revised up-and-down procedure for acute oral toxicity testing [19]. Female rats were selected for the test because they are frequently more sensitive to the toxicity of test compounds than males. The extract was dissolved in sterilized distilled water in a volume of 10 mL/kg body weight of experimental animal by oral administration. All the animals were fasted overnight, but with free access to water and they were weighed before the extract administration. Animals were randomly divided in two groups (n = 5 per group). The first group (control group) received distilled water orally. The second group (acute toxicity group) was treated as followed: a dose limit of 5 g/kg was given to the first animal which was observed for mortality, signs of acute toxicity and behavioral changes (unusual aggressiveness, unusual vocalization, restlessness, sedation and somnolence, twitch, tremor, ataxia, catatonia, paralysis, convulsion, fasciculation, prostration and unusual locomotion and asphyxia) for the first thirty minutes and the first hour, then hourly for 5 h and, finally periodically until 48 h. If this first animal survived, then four additional animals were to be given the same 5 g/kg dose sequentially at 48 h intervals. All the experimental animals were individually observed daily for general behavioral and body weight changes, hazardous symptoms and mortality for a period of 14 days post treatment. The LD50 was predicted to be above 5 g/kg if three or more rats survived. At the end of the experimental period, all animals were weighed and sacrificed by cervical dislocation, and the organs were excised for necropsy.

### Sub-chronic toxicity

#### Experimental design

The administered doses in this study were chosen based on the pharmacologically effective doses of the hydroethanolic extract from earlier hepatoprotective effects studies carried out in our laboratory in males Wistar rats. A total of 40 rats of either sex were used in this study. Rats were divided into 4 groups of 10 each (n = 10; 5 males and 5 females per group) and their weights were recorded. Before treatment, rats were individually handled and carefully examined for abnormal behavior and appearance. The extract of *C. capitatum*, dissolved in sterilized distilled water, was administered orally once a day for 28 consecutive days in single doses of 0.4 g/kg (group I), 0.8 g/kg (group II) or 1.6 g/kg (group III), while the control rats (group IV) received sterilized distilled water. Animals were observed daily during the experimental period for mortality or morbidity, changes in posture, changes in skin fur, eyes, mucous membranes and behaviors. At the end of each week, the body weights of all the rats were recorded and doses of the extract were adjusted accordingly. At the end of the 28 days of administration, animals were fasted overnight, but allowed free access to water. On the 29th day they were anesthetized with ether and blood samples were collected via retro-orbital puncture using capillary tubes for hematological and biochemical studies respectively [20]. Blood samples for hematological analysis were collected in tubes containing Ethylene Diamine Tetra Acetic acid (EDTA) as anticoagulant whereas samples for biochemical analysis were collected into tubes without anticoagulant and centrifuged within one hour. After blood collection, the rats were sacrificed by clavicle dislocation. Organs such as liver, kidneys, sex organs (testes, seminal vesicles and epididymis for male rats; ovaries and uterus for female rats), brain, spleen, lungs, and heart were excised, washed immediately in NaCl (0.9%), weighed individually and examined macroscopically.

#### Relative organ weights ratio

The excised organs were weighed and the weight of each organ was multiplied by 100 and divided by the weight of the animal before sacrifice to obtain the relative organs weight (%) [21, 22].

#### Hematological and biochemical analysis

Blood samples collected in EDTA tubes were used for hematological analysis. Parameters included: red blood cell count, white blood cell count, hemoglobin, hematocrit, mean corpuscular volume, mean corpuscular hemoglobin, mean corpuscular hemoglobin concentration, platelets count and mean platelet volume were performed using an automatic hematological analyzer (ABX Pentra XL 80, France). The differential leukocyte count was performed with an optical microscopy (Optika, Italy) after hematological staining (fixation with May Grunwald and staining with Giemsa stain (Atom Scientific, UK)). In each case, 100 cells were counted. Blood samples collected in tubes without anticoagulant were used for biochemical analysis. They were allowed to clot and centrifuged at 3000 rpm corresponding to 1107g force for 15 min using electric centrifuge (Shimadzu Scientific Corporation Tokyo, Japan) within 1 h after collection. The sera were separated, stored at -20°C and used for evaluation of biochemical parameters which include aspartate aminotransferase (AST), alanine aminotransferase (ALT), alkaline phosphatase (ALP), creatinine, glucose, serum urea nitrogen, uric acid, total cholesterol, triglycerides, total protein, total bilirubin and direct bilirubin, a-amylases, chloride, potassium, sodium and inorganic phosphorus using commercial kits purchased from Human GmbH. D-65205, Wiesbaden, Germany.

### Statistical analysis

The results are expressed as mean ± standard error of the mean (SEM). Statistical analysis was performed by one-way analysis of variance (ANOVA) followed by Tukey's multiple comparison test to evaluate significant differences between groups. Results were considered to be significant at p < 0.05. All statistical analyses were performed using GraphPad Prism 5 Software Inc., USA.

## Results

In acute toxicity study, oral administration of hydroethanolic leaf extract of *C. capitatum* at the dose of 5 g/kg did not cause mortality or any clinical signs of acute toxicity in rats observed for a short period of 48 h and a long period of 14 days. All five female rats survived until the end of the observation period. No abnormality was found in organs at necropsy.

### Sub-chronic toxicity

**Figure 1:**
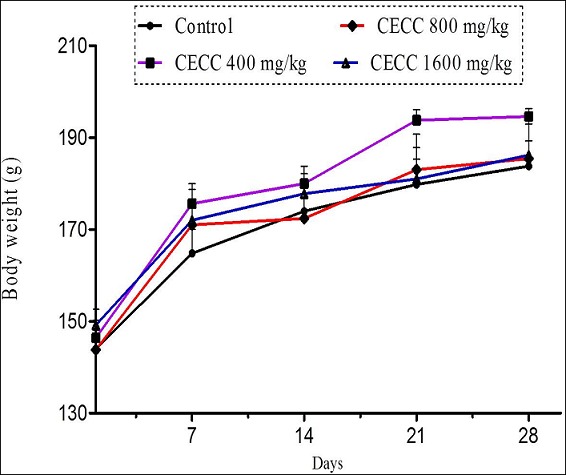
Mean body weight gain in sub-chronic toxicity of the hydroethanolic leaf extract of Clerodendrum capitatum in male rats

**Figure 2:**
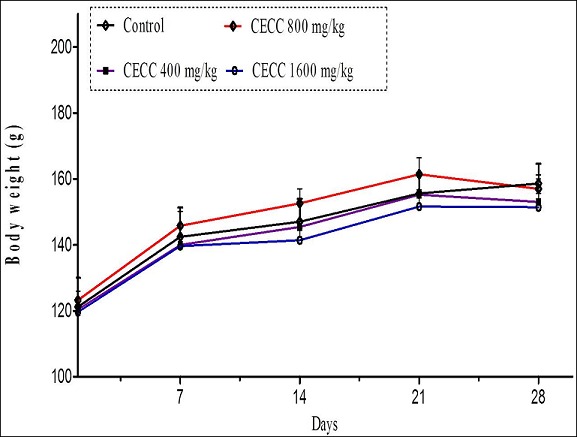
Mean body weight gain in sub-chronic toxicity of the hydroethanolic leaf extract of Clerodendrum capitatum in female rats

In sub-chronic toxicity study, daily oral administration of the hydroethanolic leaf extract of *C. capitatum* at the doses of 0.4 g/kg, 0.8 g/kg and 1.6 g/kg for 28 consecutive days resulted in no noticeable changes in the general behavior of treated rats compared to controls. No lethality was recorded in either sex in control and treated groups at any of the doses administered during the 28 days of extract administration. No differences in food and water consumption were observed between the groups of rats. Likewise, no significant differences in body weight gain (Figure 1 and Figure 2) or relative organ weights (Table 1, Table 2) were observed between control and treated groups. Macroscopic observations of organs of treated animals did not show any significant change in color and texture between control and treated groups. Both the control and treated rats appeared uniformly healthy at the end of and throughout the 28-day treatment period. Similarly, sub-chronic oral administration of the extract did not cause any significant change in hematological profile of male and female rats (Table 3, Table 4). However, white blood cells and hemoglobin rate increased in treated animals. The extract did not cause any significant change in serum creatinine, total and conjugated bilirubins, total proteins, triglycerides, phosphorus, potassium, chlorine, sodium and uric acid in both male and female rats (Table 5, Table 6). However, at 1.6 g/kg the extract decreased significantly (p < 0.05) blood concentration of urea in both male and female rats as compared to control. In the same way, total cholesterol and glucose concentrations decreased significantly in both male and female rats. Liver enzymes (AST, ALT and ALP) also decreased in treated animals (Table 5, Table 6).

**Table 1:** Effect of CECC on mean relative organ weight (%) in male rats

Organs	Control	Extract doses (mg/kg/day)		
		400	800	1600
Liver	3.59 ± 0.28	3.35 ± 0.14	3.27 ± 0.17	3.29 ± 0.09
Kidney (right)	0.45 ± 0.02	0.38 ± 0.03	0.39 ± 0.02	0.41 ± 0.03
Kidney (left)	0.45 ± 0.01	0.39 ± 0.02	0.40 ± 0.02	0.41 ± 0.02
lungs	0.65 ± 0.05	0.55 ± 0.04	0.54 ± 0.03	0.64 ± 0.03
Spleen	0.29 ± 0.02	0.27 ± 0.02	0.22 ± 0.01	0.27 ±0 .01
Heart	0.34 ± 0.02	0.33 ± 0.01	0.34 ± 0.01	0.34 ± 0.01
Testis (right)	0.76 ± 0.03	0.69 ± 0.03	0.46 ± 0.13	0.73 ± 0.03
Testis (left)	0.75 ± 0.04	0.68 ± 0.02	0.46 ± 0.12	0.74 ± 0.03
Epididymis	0.40 ± 0.02	0.36 ± 0.04	0.29 ± 0.05	0.32 ± 0.04
Brain	0.99 ± 0.07	0.84 ± 0.07	0.84 ± 0.05	0.99 ± 0.06
Seminal vesicle	0.85 ± 0.02	0.65 ± 0.05	0.76 ± 0.05	0.89 ± 0.09

Values are expressed as Mean ± SEM (n = 5)

**Table 2:** Effect of CECC on mean relative organ weight (%) in female rats

Organs	Control	Extract doses (mg/kg/day)		
		400	800	1600
Liver	3.53 ± 0.18	3.20 ± 0.20	3.36 ± 0.12	3.90 ± 0.14
Kidney (right)	0.47 ± 0.02	0.41 ± 0.01	0.43 ± 0.01	0.44 ± 0.01
Kidney (left)	0.45 ± 0.02	0.41 ± 0.02	0.45 ± 0.01	0.45 ± 0.02
lungs	0.79 ± 0.03	0.77 ± 0.07	0.69 ± 0.02	0.83 ± 0.04
Spleen	0.34 ± 0.01	0.29 ± 0.02	0.30 ± 0.01	0.33 ± 0.01
Heart	0.40 ± 0.01	0.40 ± 0.02	0.36 ± 0.01	0.36 ± 0.00
Ovary	0.05 ± 0.01	0.05 ± 0.01	0.04 ± 0.01	0.04 ± 0.01
Uterus	0.66 ± 0.01	0.57 ± 0.01	0.58 ± 0.01	0.63 ± 0.01
Brain	1.04 ± 0.07	0.92 ± 0.11	1.00 ± 0.07	1.13 ± 0.02

Values are expressed as Mean ± SEM (n = 5)

**Table 3:** Effect of CECC on hematological profile of male rats

Hematological parameters	Control	Extract doses (mg/kg/day)		
		400	800	1600
White blood cells (10^9^/l)	7.82 ± 1.03	9.96 ± 0.76	9.48 ± 0.57	9.74 ± 1.23
Red blood cells (10^12^/l)	8.09 ± 0.20	8.31 ± 0.11	8.36 ± 0.09	8.13 ± 0.08
Hemoglobin (g/dl)	15.88 ± 0.21	16.42 ± 0.16	15.90 ± 0.18	16.06 ± 0.14
Hematocrit (%)	44.88 ± 0.68	46.28 ± 0.59	44.46 ± 0.62	51.46 ± 5.68
MCV (fl)	55.62 ± 1.20	55.76 ± 1.09	53.22 ± 0.75	56.70 ± 0.47
MCH (pg)	19.60 ± 0.32	19.68 ± 0.23	18.96 ± 0.19	19.68 ± 0.14
MCHC (%)	35.36 ± 0.29	35.42 ± 0.32	35.72 ± 0.25	34.82 ± 0.13
Platelets (10^9^/l)	654.00 ± 31.33	672.40 ± 21.44	698.00 ± 41.06	686.8 ± 24.14
MPV (fl)	8.180 ± 0.11	8.16 ± 0.12	8.36 ± 0.040	8.00 ± 0.07
Neutrophils (%)	20.60 ± 0.60	19.40 ± 0.40	20.00 ± 0.7	21.00 ± 1.81
Eosinophils (%)	2.80 ± 0.86	1.80 ± 0.73	1.80 ± 0.91	1.80 ± 0.80
Basophils (%)	0.00 ± 0.00	0.00 ± 0.00	0.00 ± 0.00	0.00 ± 0.00
Lymphocytes (%)	74.60 ± 1.60	77.20 ± 0.66	76.80 ± 1.02	75.80 ± 1.46
Monocytes (%)	1.60 ± 0.67	1.60 ± 0.50	1.40 ± 0.74	1.80 ± 0.80

Values are expressed as Mean ± SEM (n = 5). MCV, mean corpuscular volume; MCH, mean corpuscular hemoglobin; MCHC, mean corpuscular hemoglobin concentration; MPV: mean platelet volume

**Table 4:** Effect of CECC on hematological profile of female rats

Hematological parameters	Control	Extract doses (mg/kg/day)		
		400	800	1600
White blood cells (10^9^/l)	7.76 ± 0.66	8.82 ± 1.24	8.80 ± 0.68	8.96 ± 0.72
Red blood cells (10^12^/l)	7.36 ± 0.16	7.48 ± 0.08	7.50 ± 0.09	7.46 ± 0.14
Hemoglobin (g/dl)	14.82 ± 0.18	15.30 ± 0.23	15.14 ± 0.28	15.12 ± 0.182
Hematocrit (%)	42.94 ± 0.58	43.56 ± 0.64	44.32 ± 0.80	43.74 ± 0.52
MCV (fl)	58.38 ± 0.80	58.30 ± 0.64	59.12 ± 0.72	58.78 ± 1.03
MCH (pg)	20.08 ± 0.26	20.40 ± 0.28	20.10 ± 0.23	20.22 ± 0.33
MCHC (%)	34.46 ± 0.08	35.08 ± 0.30	34.10 ± 0.13	34.50 ± 0.08
Platelets (10^9^/l)	747.00 ± 16.74	699.60 ± 23.80	694.80 ± 28.48	797.60 ± 19.83
MPV (fl)	8.06 ± 0.13	8.18 ± 0.17	8.26 ± 0.04	8.02 ± 0.07
Neutrophils (%)	19.40 ± 0.67	19.40 ± 0.67	19.80 ± 0.96	20.00 ± 0.83
Eosinophils (%)	1.80 ± 0.73	1.80 ± 0.58	1.20 ± 0.58	1.40 ± 0.40
Basophils (%)	0.00 ± 0.00	0.00 ± 0.00	0.00 ± 0.00	0.00 ± 0.00
Lymphocytes (%)	76.80 ± 1.31	77.60 ± 0.8	78.00 ± 1.18	77.00 ± 1.04
Monocytes (%)	2.00 ± 0.70	1.20 ± 0.48	1.00 ± 0.44	1.60 ± 0.50

Values are expressed as Mean ± SEM (n = 5). MCV, mean corpuscular volume; MCH, mean corpuscular hemoglobin; MCHC, mean corpuscular hemoglobin concentration; MPV, mean platelet volume

**Table 5:** Effect of CECC on serum biochemical parameters in male rats

Biochemical parameters	Control	Extract doses (mg/kg/day)		
		400	800	1600
Urea (g/l)	0.31 ± 0.01	0.27 ± 0.00	0.27 ± 0.01	0.25 ± 0.01^a^
Glucose (g/dl)	0.86 ± 0.08	0.60 ± 0.04^a^	0.54 ± 0 .04^a^	0.60 ± 0.06^a^
Creatinine (mg/l)	5.20 ± 0.20	5.40 ± 0.50	4.80 ± 0.20	4.80 ± 0.20
ALT (U/l)	158.40 ± 8.18	156.00 ± 9.09	178.80 ± 9.50	151.20 ± 8.70
ALT (U/l)	61.20 ± 5.16	55.20 ± 5.16	43.20 ± 3.49	58.80 ± 7.20
Alkaline phsophatase (U/l)	300.00 ± 18.69	230.40 ± 26.06	190.80 ± 12.92^b^	253.20 ± 21.33
Total bilirubin (mg/dl)	1.14 ± 0.13	1.19 ± 0.23	1.04 ± 0.04	0.84 ± 0.22
Direct bilirubin (mg/dl)	0.21 ± 0.03	0.19 ± 0.02	0.15 ± 0.01	0.23 ± 0.02
Total proteins (g/l)	53.84 ± 1.48	50.46 ± 2.29	53.45 ± 1.44	50.43 ± 2.92
Total cholesterol (g/l)	0.72 ± 0.04	0.60 ± 0.01	0.58 ± 0.02^a^	0.57 ± 0.02^a^
Triglycerides (g/l)	0.60 ± 0.09	0.64 ± 0.03	0.6 3± 0.06	0.53 ± 0.02
amylases (U/l)	254.80 ± 8.14	265.60 ± 9.50	243.40 ± 2.06	255.20 ± 4.64
Sodium (mmol/l)	140.40 ± 1.63	143.04 ± 1.20	143.60 ± 1.93	139.80 ± 2.53
Potassium (mmol/l)	4.50 ± 0.21	4.44 ± 0.06	4.18 ± 0.29	4.74 ± 0.27
Chlorine (mmol/l)	104.40 ± 7.71	104.00 ± 0.63	103.40 ± 1.40	103.00 ± 0.70
Phosphorus (mg/l)	42.40 ± 3.44	40.60 ± 5.52	42.40 ± 5.69	34.60 ± 2.40
Uric acid (mg/l)	17.44 ± 2.90	13.68 ± 0.72	14.71 ± 3.34	26.55 ± 3.96

Values are expressed as Mean ± SEM (n = 5)

a p< 0.05

b p< 0.01, vs control group

**Table 6:** Effect of CECC on serum biochemical parameters in female rats

Biochemical parameters	Control	Extract doses (mg/kg/day)		
		400	800	1600
Urea (g/l)	0.32 ± 0.01	0.30 ± 0.01	0.29 ± 0.01	0.23 ± 0.01^a^
Glucose (g/dl)	0.85 ± 0.01	0.61 ± 0.03^b^	0.59 ± 0.03^b^	0.60 ± 0.06^b^
Creatinine (mg/l)	5.40 ± 0.24	6.20 ± 0.20	5.10 ± 0.20	5.40 ± 0.24
ALT (U/l)	159.60 ± 7.25	147.60 ± 6.99	165.60 ± 7.96	168.00 ± 3.79
ALT (U/l)	69.60 ± 8.81	55.20 ± 2.24	42.00 ± 2.68^b^	45.60 ± 3.05^a^
Alkaline phsophatase (U/l)	196.8 ± 7.91	189.6 ± 8.81	163.2 ± 6.94^a^	168.0 ± 6.84
Total bilirubin (mg/dl)	1.06 ± 0.11	1.11 ± 0.08	0.80 ± 0.09	0.88 ± 0.21
Direct bilirubin (mg/dl)	0.25 ± 0.06	0.15 ± 0.02	0.26 ± 0.05	0.23 ± 0.05
Total proteins (g/l)	66.19 ± 3.59	57.38 ± 1.80	58.71 ± 3.03	59.30 ± 2.62
Total cholesterol (g/l)	0.79 ± 0.08	0.59 ± 0.01^a^	0.57 ± 0.02^a^	0.56 ± 0.02^a^
Triglycerides (g/l)	0.62 ± 0.04	0.73 ± 0.04	0.68 ± 0.05	0.60 ± 0.04
amylases (U/l)	187.20 ± 8.97	223.60 ± 6.56	209.00 ± 6.80	230.20 ± 8.93
Sodium (mmol/l)	142.2 ± 1.24	142.60 ± 2.04	142.00 ± 1.92	141.60 ± 1.36
Potassium (mmol/l)	5.00 ± 0.58	4.12 ± 0.25	4.68 ± 0.16	4.54 ± 0.42
Chlorine (mmol/l)	101.20 ± 1.93	102.00 ±1 .51	101.20 ± 0.73	100.80 ± 1.28
Phosphorus (mg/l)	36.40 ± 4.50	29.60 ± 2.15	38.20 ± 3.77	38.00 ± 5.71
Uric acid (mg/l)	20.00 ± 3.11	15.58 ± 1.64	23.64 ± 3.11	22.70 ± 1.44

Values are expressed as Mean ± SEM (n = 5)

a p< 0.05

b p< 0.01, vs control group

## Discussion

Medicinal plants are an important source of bioactive compounds and are used worldwide in traditional medicine for the treatment of various ailments. Although medicinal plants may have biological activities that are beneficial to humans, the potential toxicity of these bioactive substances has not been well established [22]. Moreover, despite the widespread use, few scientific studies have been undertaken to ascertain the safety and efficacy of traditional remedies [23]. Thus, the safety of these plants must be studied thoroughly to avoid their potential toxicity in human. To achieve this objective, the present investigation was performed to evaluate the possible acute toxicity and 28 days sub-chronic toxicity effects of hydroethanolic leaf extract of *C. capitatum*, used as a natural medicine in many African countries to alleviate many pathological conditions alone or in combination with many others medicinal plants organs by traditional healers in their receipt formulation. Data obtained from this study showed that *C. capitatum* in acute dose of 5 g/kg, by oral route, did not produce any sign of toxicity in general behavior or death. All animals treated with the extract survived until the end of the 14 days observation period. No abnormality was found in organs at necropsy at the end of the experimental period. This suggests that the median acute toxicity value (LD50) of the extract is above 5 g/kg body weight. According to Kennedy et al. [24], substances that present LD_50_ higher than 5 g/kg by oral route may be considered practically non-toxic. Therefore, it can be suggest that acute toxicity of the hydroethanolic leaf extract of *C. capitatum* is devoid of acute oral toxicity. Similar results were observed by Mirtes et al. [25] and Caroline et al. [26] with other plants using the same toxicological method.

A good extrapolation has been reported between toxicological insults in rats and humans; this is in contrast weaker when comparing humans and mice [27]. Therefore, oral intake of the leaf of *C. capitatum* may be considered as safe. However, in an acute oral toxicity study by Adeneye et al. [15], *C. capitatum* aqueous fresh leaves extract was documented to be non-lethal in rats at 5 g/kg but caused rapid-onset increased somatomotor activity, tachypnea, bilateral narrowing of the eyelids, tremor, piloerection, and increased feeding pattern in rats. Sub-chronic treatment did not produce any death or clinical signs of toxicity. There were no statistically significant differences (p > 0.05) in the body weight gain by the animals throughout the course of extract administration in all the doses compared with the control animals. This observation may indicate that extract did not alter the metabolic processes of the treated animals which may subsequently affect the hormones and body weight [28]. An important index to diagnose whether an organ has been exposed to injury is to calculate the organ-to-body weight ratio [22]. Hence, if rats are exposed to toxic substances the weight of the damaged organ will either increase or decrease as will the organ-to-body weight ratio. In the present study there were no significant changes in the relative weights of organs between the control and treated rats. This suggests no glossy toxic effect from the extract. Analysis of blood parameters is relevant to risk evaluation as any changes in the hematological and biochemical systems have a higher predictive value for human toxicity, when data are translated from animal studies [27]. In this study, the hematological profile of treated rats showed no significant difference with control group, except white blood cells and hemoglobin which increased in the groups treated with the hydroethanolic leaf extract of *C. capitatum*. Increase in white blood cells directly indicates the strengthening of the organism defense [29, 30]. This elevation in total leucocytes count suggests that the extract contains biologically active compounds that have the ability to boost the immune system through increasing the population of defensive white blood cells.

However, a global increase was observed in red blood cell count, hematocrit and hemoglobin concentration, implying that there may be a possible increase in erythropoiesis. Kidney functions were evaluated by means of serum urea, creatinine, sodium, potassium, chloride, uric acid and inorganic phosphorus. Increase blood creatinine is a good indicator of negative impact in kidney functions [31, 32]. Moreover, serum creatinine and urea concentrations are used for the assessment of renal sufficiency [33]. Thus, higher than normal levels of serum creatinine and urea are indications of deficiency in renal function. In the present study, there were no significant changes in blood creatinine, uric acid, sodium, potassium, chloride and inorganic phosphorus. These results suggest that the kidney functions are not altered in animals treated with the extract. However, serum urea decreased in rats treated at the dose of 1.6 g/kg. The decrease in serum urea concentration in the treated rats confirms that functioning of the kidney is normal. The increase levels of AST and ALT in the blood are associated with damage of hepatic cells [34, 35]. Emerson et al. [36] also have reported that enhancement in the level of serum proteins is an indication of tissue injury and reflection of hepatic toxicity. Significant changes in such classical enzymes as ALP, ALT and AST suggest liver impairment, since these are reliable indices of liver toxicity [37], or altered integrity of cellular membrane as well as cell lyses or death [38]. As one of the biochemical parameters analyzed, AST is normally found in the cytoplasm and mitochondria of many cells, primarily in cardiac muscle, liver and skeletal muscle. Its concentration is much lower, however, in the kidney, pancreas and erythrocytes. Therefore, an increase in the serum levels of ALT, AST, ALP or total bilirubin indicates hepatic toxicity [39]. These changes occur in the blood when the hepatic cellular permeability is changed or when necrosis and cellular injury occurs. In the present investigation, sub-chronic administration of the extract caused decrease in the levels ALT, AST and ALP. No significant difference in the levels of total proteins, total bilirubin and conjugated bilirubin was found. This suggests that the extract may not only be toxic to the liver but could have hepatoprotective effects.

Moreover, sub-chronic administration of the extract caused significant decrease in the level of glucose and total cholesterol; while there was no significant change in the levels of triglyceride although it decreased, suggesting that *C. capitatum* has hypoglycemic and hypolipidemic effects. Our data concerning glucose and total cholesterol and triglycerides are in agreement with those found by Adeneye et al. [15] who demonstrated that the fresh aqueous leaf extract of *C. capitatum* possesses hypoglycemic and hypolipidemic effects. Moreover these results confirmed the traditional use of this plant in the treatment of diabetes and obesity. In the present investigation, there was no significant change in serum level of a-amylases. The a-amylases catalyze the hydrolytic degradation of polymeric carbohydrates such as amylose, amylopectin and glycogen by cleaving 1,4-a-glucosidic bounds. Because of the sparsity of specific clinical symptoms of pancreatic diseases, a-amylases determinations are of considerable importance in pancreatic diagnostics. They are mainly used in the diagnosis and monitoring of acute pancreatitis. Hyperamylasemia does not, however, only occur with acute or in the inflammatory phase of chronic pancreatitis, but also in renal failure (reduced glomerular filtration), tumors of the lungs or ovaries, pulmonary inflammation, diseases of the salivary gland, diabetic ketoacidosis or cerebral trauma. The results of our work demonstrated that *C. capitatum* did not impair the pancreas functioning.

## Conclusion

In conclusion, we summarize that acute (5 g/kg) and sub-chronic (0.4, 0.8 and 1.6 g/kg) toxicological evaluation of the hydroethanolic leaf extract of *Clerodendrum capitatum* may be considered as relatively free of toxicity, when given orally, because it did not cause any death, lethality nor produced any remarkable hematological and biochemical adverse effects in both male and female Wistar rats. Further investigations in the areas of mutagenic, teratogenic and carcinogenic effects are however needed.

### What is known about this topic

- *Clerodendrum capitatum* exerts hypoglycemic and hypolipidemic effects.
- Some constituents of medicinal plants have been shown to be potentially toxic.
- Consumption of medicinal plants without evaluating their safety can result in unexpected or toxic effects.

### What this study adds

- *Clerodendrum capitatum* may be considered as relatively free of toxicity when administrated orally during 28 days.
- *Clerodendrum capitatum* leaf extract may contain biologically active compounds that have the ability to boost the immune.
